# A community participatory approach to enhancing capacity in patient-centered alcohol research: Bridging translational science and empowering women

**DOI:** 10.1017/cts.2025.10102

**Published:** 2025-07-16

**Authors:** Pravesh Sharma, Hannah Kolarik, Christopher Plapparampil Benny, Brianna N. Tranby, Tessa Vance, Tommi Thompson, Kasey R. Boehmer, Alanna M. Chamberlain, Christi A. Patten

**Affiliations:** 1 Mayo Clinic Health System, Eau Claire, WI, US; 2 Medical College of Wisconsin, Wausau, WI, US; 3 Mayo Clinic, Rochester, MN, US; 4 Wisconsin Women’s Health Foundation, Madison, WI, US

**Keywords:** Patient-centered, comparative effectiveness research, cer, alcohol, women

## Abstract

Alcohol misuse among women has risen compared to men. Women experience barriers to engaging in patient-centered comparative effectiveness research (CER) that, in turn, limits the evidence base for addressing alcohol misuse in this population. In this manuscript, we describe WomenWise, a community-partnered project and outline how we co-developed community focused CER training curriculum and collaboratively planned future partnered learning sessions (PLSs) with Community Advisory Board (CAB) feedback. Through this approach we aim to empower women to contribute to future patient-centered CER and enhance the stakeholder capacity for future patient-centered research.

## Introduction

Excessive use of alcohol (or alcohol misuse) [1] is a critical public health concern in the United States, contributing to significant medical and psychiatric morbidity and mortality [2–4]. A significant disparity exists among women themselves, adding to the overall complexity of alcohol misuse. According to the 2023 National Survey on Drug Use and Health (NSDUH), [5] approximately 8.7% of women in the U.S. met the criteria for alcohol use disorder (AUD) in the past year. When examined by race and ethnicity, prevalence was highest among American Indian or Alaska Native women (13.6%), followed by White (11.6%), Black (10.5%), Hispanic (10.1%), and Asian women (6.1%). Recent trends also point to a troubling rise in alcohol misuse among younger women: 31.6% of women aged 18 to 25 reported binge drinking, exceeding the rate observed in their male peers. The evidence also shows that women with lower income or educational attainment are more likely to develop alcohol-related medical conditions than those with greater socioeconomic advantage. [6] These findings highlight the role of structural inequities that are rooted in socioeconomic position and demographic factors in shaping risks and disproportionately burdening vulnerable subgroups of women.

Furthermore, longitudinal studies have documented that women experience more significant alcohol-related adverse consequences compared to men, including increased rates of emergency room visits, hospitalizations, and deaths related to alcohol misuse [7]. The complex interplay of social, logistical, and awareness-related factors can explain this emerging trend. For example, compared to men, women experience added caregiver responsibilities and are labeled negatively (“bad character”) for using alcohol. Women are often unaware of the harmful effects of alcohol specific to their sex, such as higher blood alcohol concentration compared to men with the same amount of alcohol consumption [8]. These factors independently or interactively influence women’s ability to seek and access alcohol treatment [9].

Patient-centered comparative effectiveness research (CER) focuses on research questions and outcomes that matter most to patients and their communities. It emphasizes working alongside people with lived experience—through co-learning, shared decision-making, and collaboration that starts with shaping the research question and extends all the way through how findings are shared. [10] This approach is especially important for women with alcohol misuse, given the treatment disparities they continue to face and the limited inclusion of their voices in traditional research models. [9,11]

Unfortunately, minimal comparative and patient-centered CER exists in the treatment of women’s alcohol misuse [12]. The gap stems from historical biases that have prioritized men-focused studies, as well as barriers to research participation that mirror those affecting treatment access [13]. Further, the limited use of community-engaged research among women has resulted in a lack of patient-centered methodology, which hinders the incorporation of research findings into the settings where women seek clinical care and community-based support interventions [14].

**Figure 1.**
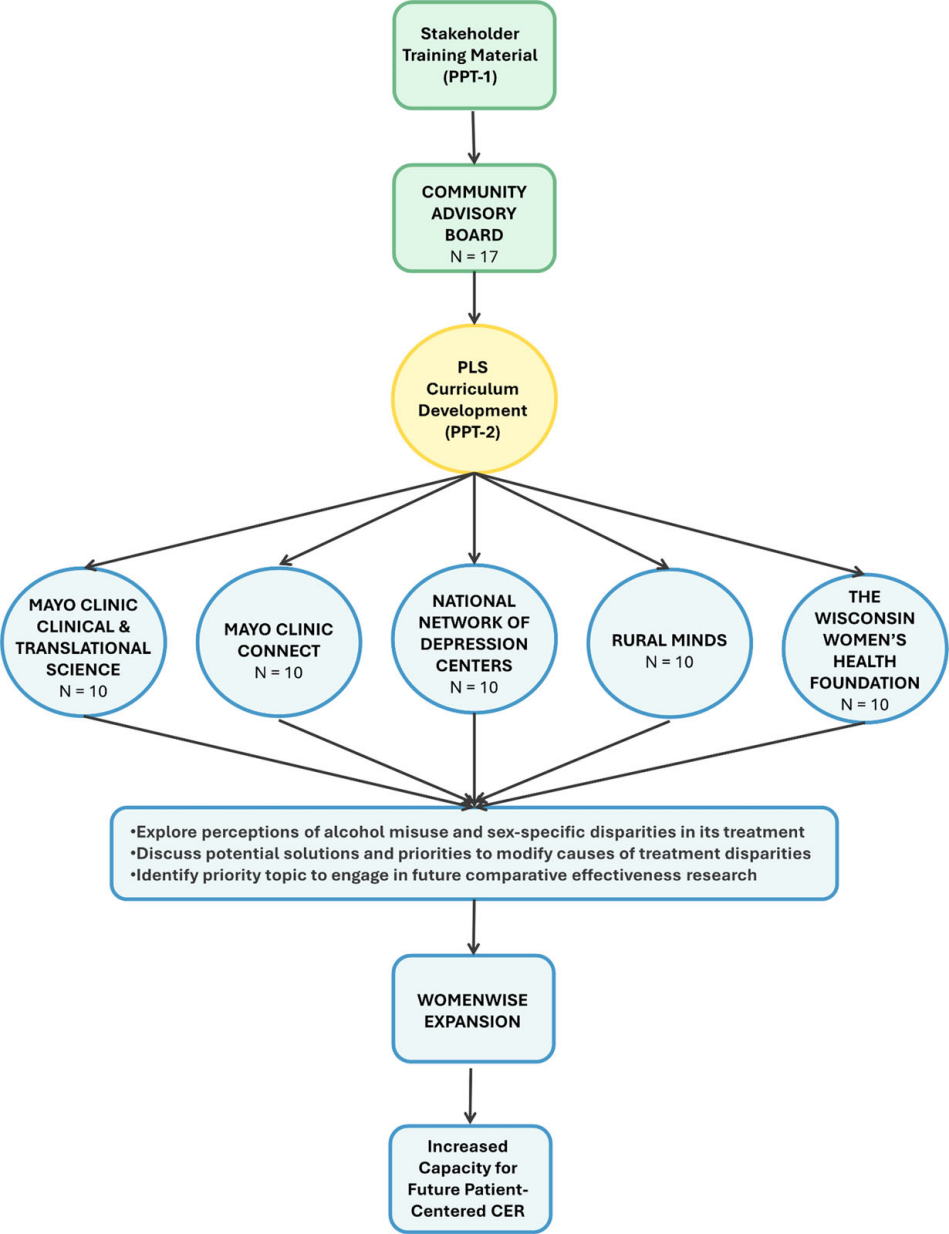
Community participatory capacity building plan for patient-centered comparative effectiveness research (CER). N: expected number of PLS attendees Abbreviations: CER=comparative effectiveness research; PLS= Partnered learning sessions; PPT= PowerPoint.

To address the underrepresentation of women in patient-centered CER on alcohol misuse treatment, our project takes a community participatory approach to build a network of women stakeholders (large non-profit organizations, faith leaders, women with lived experiences) known as *WomenWis*e (Figure 1). The objective of this network is to strengthen the capacity of women stakeholders by equipping them with knowledge of sex disparities in alcohol misuse treatment and engaging them in future patient-centered CER methodologies. Our current paper outlines this approach.

## Methods

The Mayo Clinic Institutional Review Board deemed this capacity building project “not research.”

### Project design and setting

This project was conducted at Mayo Clinic (Rochester, Minnesota) and the Mayo Clinic Health System (MCHS) in Eau Claire, Wisconsin, with the involvement of community members. The project design is highlighted in Figure 1.

### Community advisory board (CABs)

A community advisory board (CAB) of 17 women stakeholders was formed to achieve the project goals representing non-profit organizations (NPOs) across the United States (Rural Minds [15], National Grange [16], The Wisconsin Women’s Health Foundation [17], and The National Network of Depression Centers [18], faith-based organizations (churches), persons and family members with lived experience, health professionals, and Mayo Clinic Connect (Mayo Clinic Social Network [19]). Stakeholders were recruited through trusted community networks, clinical referrals, and outreach to local groups whose missions align with the goals of WomenWise.

### Stakeholder Training and Curriculum Development Process

#### Training of Stakeholders

Our project team included a panel of experts from across the Mayo Clinic enterprise with expertise in rural health, healthcare disparities, alcohol use treatment, clinical care, and patient advocacy. The panel curated the preliminary items (“PPT-1”) to educate CAB members about (1) sex-specific alcohol harms, (2) disparities in alcohol treatment access in women, and (3) foundational research concepts (including patient-centered research and CER) based on existing literature, and (4) insights from the panel’s collective experience working with diverse communities. A stepwise consensus process strategy [20] informed the refinement of the training material to ensure the inclusion of the most relevant information for the priority population. The training was delivered online to CAB members through a PowerPoint presentation via Zoom [21] by the lead author (PS) across three 90-minute sessions.

#### Curriculum Development (“PPT-2”)

Equipped with knowledge of alcohol treatment barriers in women, and with foundational understanding of patient-centered and CER, the trained CAB members further refined the original curriculum (“PPT-1”) tailored to the priority population (women with alcohol misuse) for future partnered learning sessions (PLSs). During this phase, we facilitated co-learning and elevated community partners’ insights, ensuring decisions centered on CAB members’ lived experiences and priorities. These sessions (PLSs) are scheduled to occur at each of the five stakeholder organizations (*N* = 5) and will be led by a CAB member from the respective organization. Each PLS will have ten individuals recruited from each stakeholder’s organization who represent the priority population. Therefore, *N* = 50 individuals will be *“heard and trained”* through the PLSs. This curriculum developed for PLSs particularly included common questions that patients and their families experience about patient-centered CER, e.g., (1) What are the expectations in CER, (2) How to share lived experience in CER, and (3) How to partner with researchers. The CAB members utilized their background, expertise, and knowledge to ensure the PPT-2 was succinct yet informative, culturally relevant, and aligned with the project topic. We obtained structured cognitive interview feedback on each slide of the PPT-1 through discussions and group interactions among CAB members. During the discussion, each slide was displayed, and members provided feedback on content, flow, understandability, and relevance. Members also had the opportunity to provide open-ended feedback. Participants received a $150 honorarium for each meeting. Meetings were recorded and the CAB coordinator took detailed notes. The PLSs are scheduled for May–November 2025.

### Evaluation

The senior authors (PS and CP) constructed a self-assessment questionnaire for CAB members, which was distributed to every member after each research basics session. The CAB members were required to achieve a score of 80% or better to advance to the next step. A content analysis approach [22] was used to categorize detailed notes gathered during the CAB meetings. The CAB group dynamic was self-evaluated by CAB members using a Likert scale with response options of “Agree,” “Not Sure,” and “Disagree.” In December, CAB members completed a midpoint survey assessing intervention acceptability (Acceptability of Intervention Measure, AIM; 3 items), appropriateness (Intervention Appropriateness Measure, IAM; 4 items), and feasibility (Feasibility of Intervention Measure, FIM; 4 items), using a 5-point Likert scale (1 = completely disagree to 5 = completely agree). [23].

## Results

### Quantitative results

The training session results indicated strong performance by the CAB members in both “Research Basics I” and “Research Basics II.” In both sessions, with 11 participants in each, the mean scores were 98 and 96%, respectively. Additionally, 9 participants scored 100% in “Research Basics I,” and 8 participants have a hundred percent score in “Research Basics II.” These results indicate excellent training delivery and high participant interaction. Eight CAB members completed the midpoint feedback survey which included measures of acceptability, appropriateness, and feasibility. The responses demonstrated a high level of endorsement across all three domains. The overall average score across respondents was 4.36 (range: 3.55–5.00), indicating general agreement that the WomenWise approach is acceptable, feasible, and appropriate. Item-level scores ranged from a mean of 4.13 for statements such as “*The approach is appealing to me*” and “*The approach seems easy to use*,” to 4.50 for items including “I welcome this approach,” “*The approach seems applicable*,” and “*The approach seems implementable.”*

### Qualitative results

#### Overall feedback

A key takeaway from the community-based feedback was the need to enhance the curriculum to better address stigma (e.g., removing stigmatizing words such as “AUD” and replacing it with “alcohol misuse”) while ensuring it remains concise yet comprehensive in conveying essential concepts of patient-centered CER. A great deal of discussion focused on the format of content to meet the priority population’s overall health literacy. Additionally, CAB members refined the open-ended questions for PLSs to foster a sense of empowerment and encourage non-judgmental dialogues, focusing on:

- Exploring perceptions of alcohol misuse and sex-specific disparities in treatment
- Discussing potential solutions and priorities to address the root causes of treatment disparities
- Identifying priority topics for future CER.

### Major themes

The content analysis resulted in three major themes of (1) clarity and consistency, (2) organization and flow, and (3) non-stigmatizing language (Table 1).

**Table 1.** The table demonstrates the CAB feedback and changes made to the partnered learning session curriculum

Theme	CAB Feedback	Changes Made
Clarity and Consistency	Ensure simple, clear language accessible to all. Replace “gender” with “sex” for accuracy. Standardize punctuation, fonts, and layout to match branding. Include the WomenWise logo on each slide Maintain consistency in terminology related to alcohol use.	Simplified language to an 8th-grade and less reading level. Replaced “gender” with “sex” throughout. Standardized fonts, colors, and layout to align with the logo. Added the WomenWise logo on each slide. Defined “Alcohol Misuse” at the beginning and used consistently throughout.
Organization and Flow	Add an agenda slide and structure slides logically. Use divider slides to guide transitions. Ensure a clear flow from project introduction to alcohol misuse, research gaps, treatment, research methods, and discussion. Consider including breaks.	Added an agenda slide at the beginning. Rearranged slides to follow the recommended sequence. Inserted divider slides to signal topic transitions. Adjusted flow for better comprehension and engagement.
Non-Stigmatizing Language	Avoid potentially triggering terms. Replace words like “addiction,” “disorder,” and “problem” with “misuse.” Adjust exclusion criteria language to avoid negative connotations. Remove “stakeholders” and replace them with accessible terms. Remove the figure showing women drinking alcohol	Replaced stigmatizing language with neutral alternatives. Revised exclusion criteria language to be more neutral and explanatory. Removed “stakeholders” and replaced it with terms like “community members” or “partners.” Removed figure and replaced with a neutral figure

CAB= Community Advisory Board.

#### Clarity and consistency

The most common theme was to ensure that the PLS curriculum was simple and used clear language, allowing all attendees, regardless of background, to understand the content. Given that the scope of the project is limited to biological females, it was suggested to replace “gender” with “sex” to avoid confusion regarding the characteristics of the priority population. Finally, CAB emphasized standardizing punctuation, fonts, and layout.

#### Organization and Flow

The members suggested including an agenda slide to establish expectations, followed by a logical progression from the introduction to alcohol abuse in women, research gaps, solutions through treatment, research basics, and finally, how the PLS attendees contribute to WomenWise with each section preceded by a transition slide. The CAB expressed the need to remove complex terminologies (e.g., changing “stakeholders” to “community members” or “partners,” changing “disparities” to “differences in access to care,” “disseminate and implement” to “share and put into practice” and similar phrases) and break down “busy” slides into smaller and easily conceivable bullet points. Other common feedback from CAB under this theme was to use pictures, animations, and infographics where possible to gauge attendees’ interest and assist in learning.

#### Non-Stigmatizing language

The CAB suggested the importance of eliminating language that could trigger some individuals who have a traumatic and adverse relationship with alcohol. For example, replacing stigmatizing terminologies such as “addiction,” “disorder,” and “problem” with “misuse.” In addition, “*exclusion” (*pertaining to exclusion criteria), was perceived negative, as if people are being left out unfairly. The CAB members suggested using more neutral phrasing that explains eligibility without implying rejection and suggested rewording: “Factors that make someone ineligible for a study.”

As far as the CAB group dynamic was concerned, 100% agreed that members respected each other’s opinions, and the facilitator ensured that all opinions were considered. In a CAB member feedback survey, 100% of respondents reported agreement with the WomenWise project approach (training stakeholders and conducting PLS) to increase the capacity of the priority population by educating them in patient-centered CER. The CAB responded that the WomenWise approach to educating people in patient-centered comparative research is implementable and applicable.

## Discussion

Through this community participatory project, we trained community stakeholders in patient-centered CER and empowered them to develop a new curriculum tailored for the local community members whom they represent. CAB members found the WomenWise strategy to be implementable, with high levels of acceptability, appropriateness, and feasibility. The stakeholders will take on leadership roles in their organization to further educate their members—who represent our priority population—about CER and encourage their participation in future patient-centered CER.

The CAB is not just involved in building the PLS curriculum, but each member also represents their home organizations. In this role, they are encouraged to lead within their networks, share what they’ve learned, and empower others in their organization to engage in patient-centered CER. CAB members also serve as points of contact for PLS participants from the same organization for future follow-ups. PLS attendees will be added to the WomenWise database for future CER opportunities. This structure reinforces ongoing involvement and creates a foundation for sustained engagement, knowledge sharing, and empowerment that grows from within the community. Finally, we plan to organize large-scale community events to share experiences from the WomenWise project and create new avenues for participation in patient-centered CER.

The CAB feedback (Themes 1 and 2) incorporated into the presentation material was not merely aesthetic but intentionally aimed at improving usability and actionability. Simplifying wording and improving presentation flow were key to enhancing understanding, especially for participants with limited exposure to research concepts. CAB members emphasized using plain language (at an 8th-grade reading level) and incorporating infographics to make content more accessible and shareable. They shared personal stories, noting that intellectually heavy or technical language in community-facing presentations is often poorly received. Audiences may disengage or leave early, undermining capacity-building efforts. By providing simplified, accessible materials, we aim to build the confidence of lay facilitators as they step into leadership roles. These adjustments align with the broader community-participatory goal of “meeting the community where they are,” which fosters trust, cultural humility, and a sense of ownership—factors critical to capacity-building.

Through this project, we aim to take several future steps that align with our medium-term goal of expanding WomenWise and our long-term goal of encouraging WomenWise and other community members exposed to WomenWise to engage in patient-centered CER. Ultimately, we envision that WomenWise will collaborate with other researchers in our enterprise to engage in patient-centered CER based on their interests and lived experiences.

Our project has some limitations. Firstly, there was a lack of demographic diversity in the CAB as we only had one person of color (Black), however, we included women from diverse backgrounds and various regions across the U.S. Secondly, the overwhelmingly positive response of CAB members could be due to social desirability bias [24] and a desire to align with the facilitator’s opinion. We tried to limit this bias by asking for feedback anonymously and requesting open-ended feedback as well.

This community participatory capacity-building project successfully met key milestones, including demonstrating a feasible community engagement approach to developing easy-to-understand patient-centered CER information materials for the community. In addition, the NPO representative felt empowered to take leadership roles in disseminating the knowledge acquired during this project, further strengthening the capacity of WomenWise.

Our project has practical implications. The findings offer an actionable framework for others looking to engage women and diverse community members in patient-centered CER. The stepwise, co-developed process we used can be adapted by other researchers or practitioners working in similar settings. Most importantly, it shows that when communities help draft the content, the results are not only culturally relevant but may enhance long-term sustainability.
